# Blocking of SMAD4 expression by shRNA effectively inhibits fibrogenesis of human hepatic stellate cells 

**Published:** 2015

**Authors:** Sayyad Khanizadeh, Mehrdad Ravanshad, SeyedYounes Hosseini, Parivash Davoodian, Azim Nejati Zadeh, Jamal Sarvari

**Affiliations:** 1 *Department of Virology, Faculty of Medical Sciences, Tarbiat Modares University, Tehran, Iran*; 2 *Department of Bacteriology and Virology, Shiraz University of Medical Sciences, Shiraz, Iran*; 3 *Infectious & Tropical Diseases Research Center, Hormozgan University of Medical Sciences, Bandar Abbas, Iran*; 4 *Research Center for Molecular Medicine, Hormozgan University of Medical Sciences, Bandar Abbas, Iran*

**Keywords:** SMAD4, Liver fibrosis, shRNA, Hepatic stellate cell

## Abstract

**Aim:**

In this study, to clarify the SMAD4 blocking impact on fibrosis process, we investigated its down-regulation by shRNA on activated human LX-2 cell, in vitro.

**Background:**

Liver fibrosis is a critical consequence of chronic damage to the liver that can progress toward advanced diseases, liver cirrhosis and hepatocellular carcinoma (HCC). Different SMAD proteins play as major mediators in the fibrogenesis activity of hepatic stellate cells through TGF-β pathways, but the extent of SMAD4 as a co-SMAD protein remained less clear.

**Patients and methods:**

vector expressing verified shRNA targeting human SMAD4 gene was transfected into LX-2 cells. The GFP expressing plasmid was transfected in the same manner as a control group while leptin treated cells were employed as positive controls. Subsequently, total RNA was extracted and real-time PCR was performed to measure the mRNA levels of SMAD4, COL-1A1, α-SMA, TGF-β and TIMP-1. Furthermore, trypan blue exclusion was performed to test the effect of plasmid transfection and SMAD4 shutting-down on cellular viability.

**Results:**

The results indicated that the expression of SMAD4was down-regulated following shRNA transfection intoLX-2 cells (P<0.001). The gene expression analysis of fibrotic genes in LX-2 cells showed that SMAD4 blocking by shRNA significantly reduced the expression level of fibrotic genes when compared to control plasmids (P<0.001). Vector expressing SMAD4-shRNA induced no significant cytotoxic or proliferative effects on LX-2 cells as determined by viability assay (P<0.05).

**Conclusion:**

The results of this study suggested that knockdown of SMAD4 expression in stellate cell can control the progression of fibrogenesis through TGF-β pathway blocking.

## Introduction

 Hepatic fibrosis**, **as a pathological disorder resulting in the excessive deposition of extracellular matrix is the consequence of chronic damage to the liver**. **Hepatic stellate cells (HSCs) including 5%-8% of liver tissue considered as the most impressive player in fibrosis phenotype progression. In the pathological active conditions, HSCs undergo transition stages and acquire myofibroblastic phenotype that act as the main source of fibrosis establishment. The activation of HSCs, leading to up regulation of the markers of liver fibrosis such as α-smooth muscle actin (α-SMA) and collagen I proteins(1, 2).

Accumulating evidence indicates that the TGF-β signaling pathway plays pivotal roles in hepatic stellate cells (HSCs)-mediated liver fibrogenesis (3). TGF-β, mediates its biological activity following binding to its serine/threonine kinases mediated receptors (TGF-βRI and TGF-βRII) and subsequently triggers signal transduction pathways via the downstream mediators/transcript factors, SMADs. When TGF-βRI undergoes phosphorylation by the type II receptor kinases, the activated type I receptor phosphorylates SMAD2 and SMAD3 which ultimately trigger the formation of a complex with a helper protein named SMAD4. This complex co-translocate into the nucleus where it binds to specific promoters then activate hepatic fibrosis process. Thus, as downstream transcription factors of TGF-β signaling, SMADs proteins could be the potential targets for therapeutic preventing and treatment of liver fibrosis as well as advanced liver diseases such as liver cirrhosis and HCC (3, 4). SMAD4 contains a nuclear localization signal (NLS), thus can act as a shuttle between nucleus and cytoplasm (4-6). This function in TGF- β signaling suggests that SMAD4 can be considered as a target for controlling HSCs activation during liver fibrosis. Although, the role of SMAD4 is clarified, but inhibition of its expression, as a reasonable target for fibrosis control, is controversial. In other words, the extent of its blocking effect during HSCs activation remains to be more completely elucidated. Here, to evaluate the extent and vast of SMAD4 blocking effect on HCSs activation process and fibrosis phenotype, RNA interference exploited in LX-2 cell line. Several studies have been performed on the effects of gene silencing by RNAi for the treatment of tissue fibrosis (7, 8). With respect to the central role of SMAD4 in TGF- β /SMAD signal transduction, our aim of this study was to examine in vitro if SMAD4 may be a key molecule of TGF-β signaling pathway with a role in the pathogenesis of liver fibrosis. 

## Patients and Methods

***Cell culture***

The LX-2 cells (an immortalized human HSC cell line), was kindly gifted by Professor Scott Friedman (Mount Sinai school of medicine, New York, USA). The cells were cultured in Dulbecco’s modified Eagle’s medium (DMEM, Gibco USA) were exposed with 10% fetal bovine serum (FBS, Sigma, St. Louis, USA), 100 U/ml penicillin-streptomycin (Gibco), 2 mM L-glutamine and incubated at 37ºC in 5% CO_2_ air humidified atmosphere. This condition was kept in plated cells in the negative control group, untreated cells. 

***Activation of HSCs***

To induce the trans-differentiation of LX-2 cells into activated form (fibrogenic phenotype), the LX-2 cells undergoe serum starvation and leptin treatment (a profibrogenic hormone) according to the previously described method [9]. The LX-2 cells seeded into 6 wells plate in the DMEM culture media with 0.1% FBS at 37 °C in a humidified atmosphere with 5% CO2 for 24 h. After serum starvation, HSC cultures were treated with leptin (L) (Sigma) at 100 ng/ml, which served as a positive control for proliferation, and then the cells were incubated for 48 hours. Light invert microscopy employed to phenotype assay. 

***Transfection of HSCs***

Cells were divided into 5 groups. In group A, the LX-2 cells were untreated. The B group, were LX-2 cells undergo a stress condition (leptin induction and serum starvation). In group C, the LX-2 cells were transfected with human SMAD4 shRNA expressing vector. In group D, the LX-2 cells underwent stress condition and were then transfected with SMAD4 shRNA expressing vector. Also, an empty GFP expressing plasmid (E group) was employed in a similar manner as negative control. For transfection, the LX-2 cells were seeded into 6-well plates (3×10^5^ cells/well) followed by incubation for 36 h. On the day of treatment, cultured cells that were grown to 70% confluence, were transfected with pGFP-ilenti plasmids (Applied Biological Materials (ABM) Inc) expressing SMAD4 shRNA using lipofectamine 2000 reagent in an incomplete DMEM medium, according to the manufacturer’s instructions. After transfection (approximately 6 hours), the medium was exchanged with fresh complete medium (DMEM with 10% FBS, 100 U/ml streptomycin and 100U/ml penicillin) at 37 °C in a humidified atmosphere with 5% CO_2_. Then, the plate was left for a further 48 hours. Transfection was confirmed visually by fluorescence microscopy. The targeting site of SMAD4 shRNA, includes following sequences:

5'ATTGAAAGTTTGGTAAAGAAGCTGAAGGA3'

***Cell viability by trypan Blue exclusion***

To investigate if SMAD4 blocking has a significant effect on cell viability/death, trypan Blue exclusion test under inverted microscopy was performed. To do the dye exclusion test, 100 cells were evaluated during cell counting by trypan blue vital dye and the ration of viable/clear cells divided by total cell (bleu+clear cells) and compared in different groups.

***RNA extraction and reverse transcription***

Two days after transfection, total RNA was isolated using the qiazol reagent (QIAGEN USA) according to the manufacturer’s instruction. Then reverse transcribed into cDNA using the Revert Aid First Strand cDNA Synthesis Kit (Thermo Fisher Scientific, USA). QRT-PCR was performed according to the protocol of SYBR Premix Ex Taq RT-PCR Kit (TakaraBio Inc, Japan) in applied Biosystems StepOne™ Instrument (USA). 

***Real-time PCR for expression analysis***

The primer pairs for SMAD4, α-SMA, TIMP1, TGF- β1, COL1A1 and GAPDH (Fajardy et al. BMC Molecular Biology 2009) designed by NCBI Primer-BLAST online software and as listed in (Table 1). 

The human GAPDH used as a reference gene for normalization, H_2_O as a negative control of reactions and RNA extract as a negative control of cDNA synthesis enrolled in all PCR runs to make the experiment as valid and reliable as possible. The reaction mixtures prepared according to recommended protocol. Amplification reaction performed in a 35 cycles of 20 seconds at 94°C for denaturation and 20 seconds at 60°C for annealing and extension steps. After each PCR run, melting curve analysis and gel electrophoresis was carried out to confirm specific amplification of targets. Amplification signals for different samples were normalized to the respective GAPDH signals. Then delta-delta CT (2-CT) method applied for comparing mRNA levels of tests versus control which finally represented as fold change (relative quantification).

***Statistical analysis***

All data that presented in the results section belonged to at least three independent experiments. Statistical analysis was performed using Graph Pad Prism software. They were analyzed using one-way ANOVA to evaluate the difference between the mediums. The statistical significance between controls and treated groups was evaluated further by Tukey post-test. A P value of 0.05 was considered statistically significant.

## Results

***No significant viability change after*** ***SMAD4*** ***blocking***

Altogether visual detection and repetitive trypan blue exclusion revealed that the viability in five different groups was not significantly different even between transfected and un-transfected cells. Our data collectively indicated no significant survival difference between groups by our primary analysis (P-value<0.05). 

***SMAD4*** ***is down-regulated in*** ***SMAD4*** ***shRNA – treated LX-2 cells***

**Figure 1:**
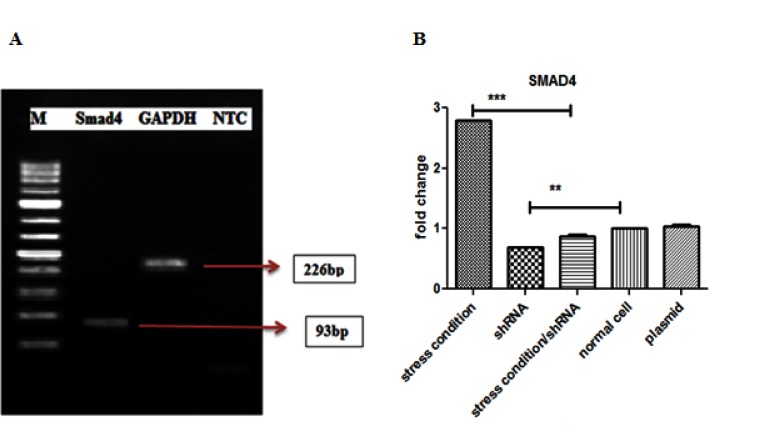
Gel electrophoresis and SMAD4 mRNA level in different groups. A-Agarose gel electrophoresis of qRT-PCR amplification of SMAD4 gene. B-The effects of SMAD4-shRNAon expression of SMAD4in LX-2 cells treated. Two days after transfection the Real-time PCR analysis showed a lower expression of SMAD4 in LX-2 cells treated with shRNA compare to control groups. M=50bp marker. Each bar is representative of the mean±standard deviation from at least three measurements and final results enrolled. ** P<0.01, *** P<0.001 showed significant levels compared to the positive control group (stress condition).

**Table 1:** The PCR primers used in this study

**Size**	**Primer pair**	**Gene name**
226	GAAGGTGAAGGTCGGAGTC(forward)	GAPDH
	GAAGATGGTGATGGGATT TC (reverse)	
93	AAGCCATTGAGAGAGCAAGGT (forward)	SMAD4
	GGTCACTAAGGCACCTGACC (reverse)	
202	TACTTCCACAGGTCCCACAAC(forward)	TIMP1
	GTTTGCAGGGGATGGATAAAC (reverse)	
147	GACAATGGCTCTGGGCTCTG (forward)	α-SMA
	CTGTGCTTCGTCACCCACG (reverse)	
123	CAGATCACGTCATCGCACAAC (forward)	TGF- β1
	GAGCAACACGGGTTCAGGTA (reverse)	
140	GAGGGCCAAGACGAAGACATC (forward)	COL1A1
	CAGATCACGTCATCGCACAAC(reverse)	

The expression of SMAD4 in the shRNA-treated LX-2 cells was evaluated by qRT-PCR (Figure 1A). The data showed that the expression of SMAD4 in shRNA treated cells was decreased significantly when compared to the normal cell (untreated) and stress condition (serum starvation/leptin-treated cells) (P<0.001). The expression level of SMAD4 mRNA in shRNA-treated cells showed lower than half of the expression in normal cells. There was also a significant difference between the expression of SMAD4 in the shRNA received cells compared with GFP- plasmid received cells as depicted in (Figure 1B) (P<0.001).

***SMAD4*** ***blocking by shRNA was able to ameliorate fibrotic effects*** ***.***

**Figure 2:**
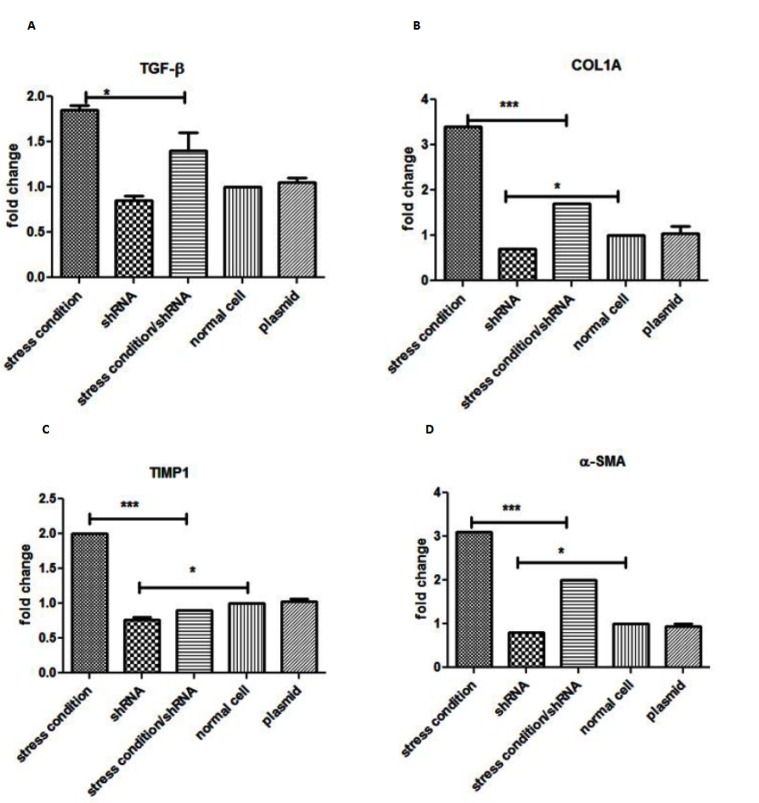
The effects of SMAD4 expression blocking on mRNA level of TGF- β1 (Figure 2A), COL1A1 (Figure 2B), TIMP-1(Figure 2C) and α-SMA (Figure 2D), that was evaluated by real-time PCR. Each bar is representative of the mean for at least 3 experiments and presented the fold increase/decrease compared to control cells expression. * P<0.05 , ** P<0.01 and *** P<0.001 indicated the significant fold changes of test groups with activated cells, empty GFP plasmid and with normal LX-2 cells for A, B and C graphs analysis

 The mRNA expression levels of pro-fibrotic genes, including TGF- β1 (Figure 2A), COL1A1 (Figure 2B), TIMP-1(Figure 2C) and α-SMA (Figure 2D), were measured by real-time PCR after exposure of activated LX-2 cells, with the SMAD4 shRNA. In the present experiment, serum starved and leptin were used to induce the fibrotic effect on the HSCs. In this study, cells were divided into groups A, B, C, D and E which were untreated LX-2 cells, LX-2 cells receiving fibrotic induction, LX-2 cells that were transfected with a vector expressing SMAD4-shRNA, the LX-2 cells that were transfected with a vector expressing SMAD4-shRNA and underwent fibrotic induction and the LX-2 cells which were transfected with empty GFP plasmid respectively. The results showed that the mRNA expression levels of collagen I, α-SMA, TIMP1 and TGF- β1 in the B group were increased by 3.5, 3.2, 1.9 and 2.2 (fold change) respectively, when compared with group A. In the D group, the mRNA expression levels of collagen I, α-SMA, TIMP1, and TGF- β1 were increased by 1.3, 1.1, 1.2 and 1.6 (fold change), respectively, compared to group C. When compared with group B, the mRNA expressions of collagen I, α-SMA, TIMP1 and TGF- β1 in the D group were decreased by 3.8, 3.4, 2.1 and 1.7 (fold change), respectively (Figure 2). Also, the results showed no significance difference between new group A and new group E (Figure 2), indicating that plasmid transfection did not affect target genes expression (P>0.05). Data showed a significant decrement of mentioned fibrotic markers in the group receiving shRNA in comparison to control groups. 

## Discussion

Liver fibrosis is the excessive accumulation of extracellular matrix proteins in tissue during chronic tissue injury. Liver fibrosis is a common pathway in all advanced chronic liver diseases, irrespective of the underlying etiology. Hepatic stellate cells and TGF-β1 cytokine seem to be the central performer in fibrosis orchestra. TGF-β1 related signalling pathway activates hepatic stellate cells then prompts their transition into myofibroblast cells phenotype (a key event in fibrosis development). Signal transduction of TGF-β1 depends on a collection of mediators, SMAD proteins, that following multimerization migrate into nucleus and translate the activation signal into gene expression level (5, 10). 

Since no approved therapeutic approach for liver fibrosis has been established, the exploration of novel therapeutic drugs as well as the investigation of key molecular targets for control of fibrosis is demanding. According to different studies, RNA silencing through RNAi has been already shown to be effective (7, 8, 11). In the present study, SMAD4-shRNA was used for inhibition of activation and fibrogenesis of HSCs. Results have shown that fibrotic gene expressions in treated LX-2 cells with shRNA were significantly decreased (P<0.001) when compared with those in untreated LX-2 cells following induction with leptin. This data shows that SMAD4 plays a critical role in induced fibrosis. Employment of RNA interference in various studies has shown antifibrotic effects on tissue fibrosis (8, 12). The results of a study showed that blockade of TGF-β signaling by TGFβRII siRNA can lead to the inhibition of metastasis, and proliferation while induced apoptosis in adenocarcinomic cell lines (13). Also, it has been reported that diminish of SMAD3 expression by siRNA could markedly hinder the liver fibrosis via disruption of TGF-β signaling (14). In our study transfection of the LX-2 cells by vector expressing SMAD4-shRNA resulted in not significant induced apoptosis. In their study, wang et al. showed that down-regulation of SMAD3 could impair the deposition of extracellular matrix protein in skin scars (14).

SMAD4 as a coordinator SMAD protein plays an important role during TGF-β1 signal transduction, as it can bind to the activated SMAD molecules such as type 2 and 3 SMAD molecules. Following the formation of a multimeric complex of SMAD2, 3 and 4, which then translocate into the nucleus, dozens of fibrotic markers up-regulated as the final event of TGF-β signaling pathway (15). Thus, impairment of the SMAD4 expression may be valuable for the treatment of tissue fibrosis (16). Our data demonstrate the mRNA expression of SMAD4 in the LX-2 cells transfected with vector carrying SMAD4-shRNA was markedly reduced (P<0.001), which indicates SMAD4 is significantly down-regulated. Morishita et al. reported that SMAD4 knockdown by specific siRNA is one of the therapeutic tools for the control of renal fibrosis in vivo (17). It has been shown that SMAD4 knockout mice impairs TGF-β/SMAD3 signal transduction, subsequently triggers modulation of multifunctional roles of TGF-β1 in fibrogenesis via interacting with SMAD transcription factors, including SMAD7 and SMAD3 to impact their transcriptional activities in renal fibrosis (18). The results of our study suggest that SMAD4-shRNA is able to reduce HSC-mediate fibrogenesis via down-regulation of hepatic fibrosis markers especially col1, α-sma and TIMP1. Our data is similar to other studies (19-21). The results obtained from a study on liver fibrosis and HCC in mice injected with CCL_4_/ethanol has shown that disruption of SMAD4 can delay the development of hepatic fibrosis and liver cancer via decreased expression of collagen 1 and SMAD4 (19). Hernanda et al. demonstrated that SMAD4 represent a tumor-driving role in HCC using SMAD4-shRNA in a sub-population of HCC tumors. During their study, Hernanda et al. observed a significant elevation of nuclear SMAD4 that can be considered as an outcome predictor and a therapeutic target (20). In addition, gene silencing using SMAD4-shRNA has shown that SMAD4 knockdown during fibro genesis of C2C12 myoblasts may become a therapeutic target for the management of skeletal muscle fibrosis (21). Altogether, these results collected with previous results, suggest that SMAD4 blocking could be one of the effective therapeutic approaches to inhibit the expression of myofibroblasts resulting in tissue fibrosis.

In conclusion, our result indicates that SMAD4 knockdown by SMAD4-shRNA can lead to the decreased expression of fibrotic genes. These results also suggest that disruption of TGF-β1 signal transduction can be considered as one of the critical therapeutic strategy for the treatment of liver fibrosis and prevent advanced liver diseases such as end stage liver cirrhosis and HCC. This data also highlighted SMAD4 as a suitable target for gene therapy and again emphasized TGF-β signaling pathway.
